# Resequencing of 558 Chinese mungbean landraces identifies genetic loci associated with key agronomic traits

**DOI:** 10.3389/fpls.2022.1043784

**Published:** 2022-10-12

**Authors:** Xuesong Han, Li Li, Hongwei Chen, Liangjun Liu, Longqin Sun, Xingmin Wang, Yantao Xiang, Zhenghuang Wan, Changyan Liu

**Affiliations:** ^1^ Institute of Food Crops, Hubei Academy of Agricultural Sciences/Hubei Key Laboratory of Food Crop Germplasm and Genetic, Wuhan, China; ^2^ College of Agronomy, Yangtze University, Jingzhou, China

**Keywords:** mungbean, landraces, resequencing, agronomic traits, gwas

## Abstract

Mungbean is a warm-season annual food legume and plays important role in supplying food and nutritional security in many tropical countries. However, the genetic basis of its agronomic traits remains poorly understood. Therefore, we resequenced 558 Chinese mungbean landraces and produced a comprehensive map of mungbean genomic variation. We phenotyped all landraces in six different environments. Genome-wide association studies (GWAS) produced 110 signals significantly associated with nine agronomic traits, for which several candidate genes were identified. Overall, this study provides new insight into the genetic architecture of mungbean agronomic traits. Moreover, the genome-wide variations identified here should be valuable resources for future breeding studies of this important food legume.

## Introduction

Mungbean (*Vigna radiata* L.) is one of the most important warm-season legume crops. Rich in essential amino acids, vitamins, and micronutrients, mungbean offers many benefits to human health (Nair et al., 2013). Because of its excellent nutritional content, short crop duration, and ability to fix nitrogen, mungbean is widely cultivated as a whole food in South, East, and Southeast Asia, particularly India and China (Graham and Vance, 2003). As for other crop species, breeding has led to a reduction of mungbean genetic diversity (Smýkal et al., 2018). However, individual landraces have evolved from their wild progenitor under natural and human selection, leading to the maintenance of relatively high genetic diversity across the species. Mining and utilization of genetic variability in landraces are important ways to widen the genetic bases of modern varieties and enable breeders to develop genetic solutions to new or existing challenges of crop production practices (Varshney et al., 2020). Despite the status of mungbean as an important leguminous food source with a highly diverse landrace germplasm (Schafleitner et al., 2015), genomic information for molecular breeding, studies of genetic diversity, and genetic mapping are lacking in this species (Isemura et al., 2012; Kim et al., 2015). Identifying the genetic basis of mungbean’s diverse landraces will provide important insight to facilitate the breeding of elite varieties for sustainable agriculture.

Detecting genetic factors that contribute to yield and quality on a genome-wide scale is crucial for crop improvement. One powerful approach to identifying genes or quantitative trait loci (QTLs) underlying complex traits is the genome-wide association study (GWAS) (Liu and Yan, 2019). This strategy has been successfully applied to a broad range of crops, including rice (Huang et al., 2010; Li et al., 2020), maize (Xiao et al., 2017), wheat (Wang et al., 2020), soybean (Fang et al., 2017), cotton (Ma et al., 2018), and food legumes (Varshney et al., 2017; Varshney et al., 2019; Wu et al., 2020). Rapid advances in high-throughput sequencing technologies and the completion of the mungbean reference genome sequence (Kang et al., 2014) have enabled the detection of genomic variation in a large-scale collection of mungbean accessions. Several studies have utilized genotyping by sequencing (GBS) to investigate population structure in mungbean (Noble et al., 2017; Breria et al., 2020; Ha et al., 2021). Loci associated with variation in mungbean seed coat color (Noble et al., 2017) and seed coat luster (Breria et al., 2020) were identified through GWAS. Recently, 2,912 SNPs and 259 gene PAV events associated with 33 agronomic traits were revealed by GWAS in mungbean (Liu et al., 2022). So far, there are no studies have focused on phenotyping diverse mungbean landraces from different environments. In addition, GWAS approaches have not been applied to the analysis genetic architecture and favorable allele usage of complex agronomic traits in a large collection of Chinese mungbean landraces.

In this study, we aimed to better understand the genetic basis of agronomic traits and genetic variability in mungbean landraces from geographically diverse regions in China. To this end, we resequenced 558 accessions with 9.83-fold coverage depth. We analyzed genomic variation, evaluated phenotypic variation across six environments, and performed GWAS for nine agronomic traits. Our results present a collection of genes or alleles that may be helpful for enhancing the genetic diversity of mungbean varieties, and provide valuable genomic information for future mungbean breeding programs.

## Materials and methods

### Plant materials and phenotyping

Five hundred and fifty-eight Chinese mungbean landraces were used for resequencing and phenotypic data collection. The accessions were selected on the basis of the germplasm database records of geographic origin and phenotypic variation to maximize genetic diversity. These mungbean lines are from 16 provinces in China, spanning most of the geographic range of mungbean. Detailed information of the 558 accessions is listed in **Table S1**.

For phenotyping, all accessions were grown in six natural environments at four different locations in 2019 to 2021: Ezhou (30.40° N, 114.89° E), Hubei province, in 2019 (2019_EZ) and 2021 (2021_EZ); Lingshui (18.50° N, 110.04° E), Hainan province, in 2019 (2019_LS) and 2020 (2020_LS); Wuhan (30.58° N, 114.03° E), Hubei province, in 2021 (2021_WH); and Gucheng (32.29° N, 111.52° E), Hubei province, in 2021 (2021_GC). In 2019_LS and 2020_LS, all accessions were sown in late October and harvested in early January of the following year, in the tropical environment of Lingshui on Hainan Island. All accessions planted in 2021_WH were sown in mid-April (spring growing season) and harvested in early July. In the other three environments, the 558 landraces were planted in summertime at different planting dates from early to late June, with harvests in October. Two replicates were performed in each environment. Each plot in the six environments contained one row 2 m in length, with 11 plants per row, 20 cm between plants within each row, and 30 cm between rows. Phenotypic trait data were collected according to a quantitative and descriptive method previously published for descriptors and data standards (Cheng, 2006). All 11 individual plants from each plot and each accession were used to measure the seven yield-related traits. Several traits were not investigated in all six environments, owing to resource limitations (**Table S6**). The seeds of each accession harvested from the 2019_EZ environment were used for protein and starch content measurements by near-infrared reflectance spectroscopy analysis. Phenotypic data for each accession used in subsequent analyses was defined as the average of the two replicates in the same environment.

### DNA extraction and sequencing

For each accession, genomic DNA was extracted from young leaves of a single two-week-old plant using the cetyltrimethylammonium bromide (CTAB) method (Chen and Ronald, 1999). At least 5 µg of genomic DNA from each accession was used to construct a sequencing library following the manufacturer’s instructions (BGI Shenzhen, China). The libraries, with an insert size of approximately 500 bp, were sequenced on a MGISEQ-2000 sequencer, generating 150 bp paired-end reads. Raw reads were cleaned using SOAPnuke v2.0.5 (Chen et al., 2018) to remove residual adaptor sequences and reads with low-quality scores.

### Variant calling and annotation

Cleaned reads for each accession were mapped to the mungbean reference genome using BWA v0.7.17 (Li and Durbin, 2009; Kang et al., 2014) with default parameters to obtain SAM files. SAMtools v1.9 (Li et al., 2009) was used to convert SAM files into BAM and sort them. The sorted files were processed by duplicate marking and indexing using the MarkDuplicates tool in GATK v4.1.8 (McKenna et al., 2010) and SAMtools, respectively. The HaplotypeCaller tool in GATK was then used with default parameters to generate GVCF files for each accession. After all GVCF files were merged, a raw population genotype file was created using GenotypeGVCFs in GATK with default parameters.

To ensure accuracy of the variants, we performed a two-step filter. Firstly, hard filtering was applied to the raw variant set using VariantFiltration in GATK, with parameters ‘QD < 2.0 || MQ < 40.0 || FS > 60.0 || SOR > 3.0 || MQRankSum < -12.5 || ReadPosRankSum < -8.0’ applied to SNPs, and ‘QD < 2.0 || FS > 200.0 || SOR > 10.0 || MQRankSum < -12.5 || ReadPosRankSum < -8.0’ applied to indels. Secondly, the variant set output from GATK was further filtered using PLINK v1.9 (Chang et al., 2015) with the minor allele frequency set to >0.05 and missing rate of <0.2. Beagle v5.2 (Browning et al., 2018) was used for missing data imputation. The variants in the small scaffolds were then removed. SNPs located in the 11 pseudomolecules of the mungbean reference genome as the final variant data set and used for subsequent population genetic analyses and GWAS. The identified SNPs and indels were further annotated with ANNOVAR (Wang et al., 2010) and were grouped on the basis of mungbean genome annotation information.

### Genome-wide association analysis

The final variant data set of the entire population was used for GWAS. Kinship matrices of relatedness between the accessions were calculated using the “-gk” function of Genome-wide Efficient Mixed-Model Association (GEMMA). These kinship matrices were then used to correct the population structure. The association analysis was performed using GEMMA, which was designed to handle large dataset analysis. The qqman R package (Turner, 2014) was used to generate quantile-quantile and Manhattan plots from GEMMA results files. The genome-wide significance thresholds of all tested traits were set as 1/*n* (*n* = total SNP number used in the association analysis).

## Results

### Genome variation map

**Figure 1:**
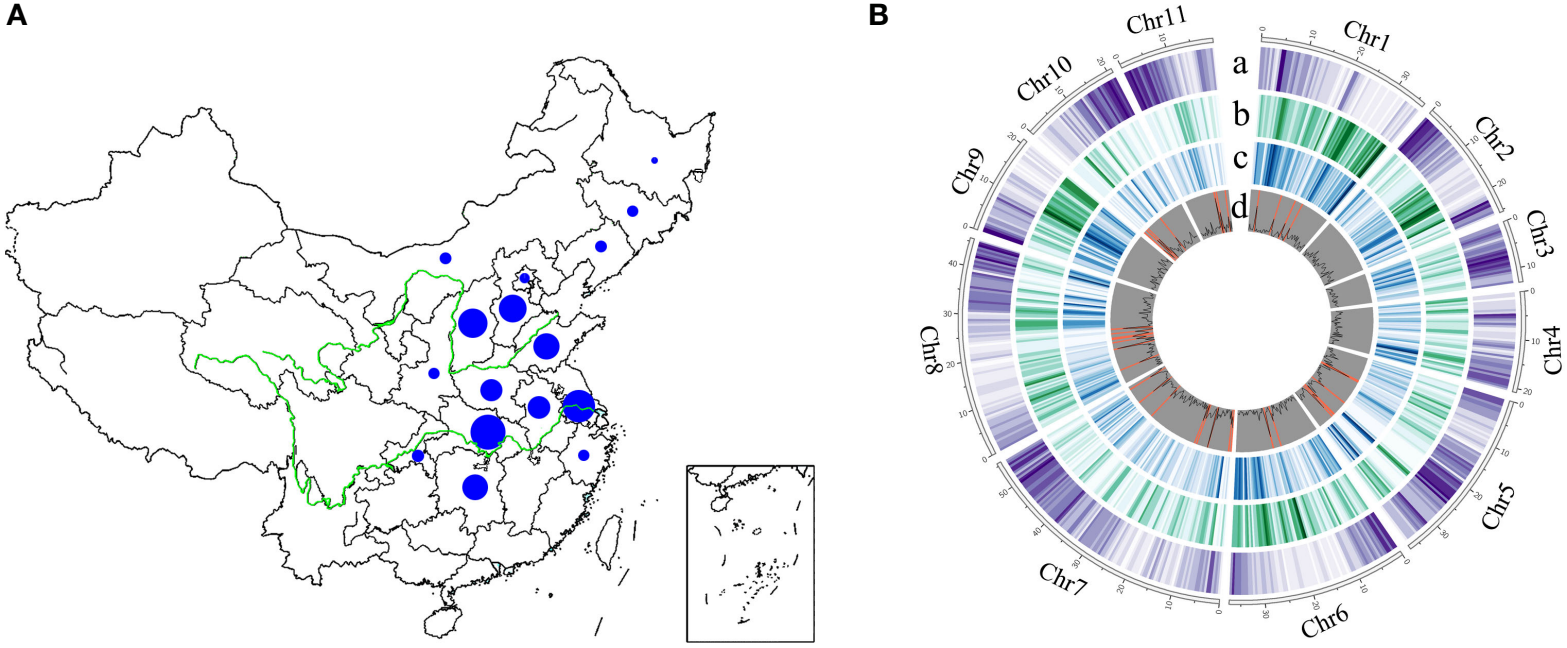
The geographical distribution and genome-wide variations of 558 mungbean landraces. The size of the blue pie represents the number of samples. **(A)** Landraces collected from 16 provinces in China. **(B)** A total of 2,582,180 high-quality SNPs and 412,999 indels (ranging from 1 to 244 bp in length) were obtained among 558 landraces across the 11 mungbean chromosomes. Track a, Gene density; b, SNP density; c, indel density; d, genomic regions with ratio of non-synonymous to synonymous SNPs > 2.5 (highlighted in orange).

To generates a comprehensive genome variation map in mungbean, we resequenced a total of 558 Chinese mungbean landraces selected from 16 provinces in North China, the Huang-Huai-Hai region, and the Yangtze River region (**Figure 1A** and **Table S1**). Approximately 16.93 billion 150-bp paired-end reads (2.54 Tb clean data) were generated, resulting in 97.10% of reads mapped and 89.93% genome coverage. The average sequencing depth was 9.83-fold, ranging from 5.97- to 19.19-fold, based on the mungbean *VC1973A* reference genome (Kang et al., 2014). Genotype coverages at six-, 12- and 18-fold averaged 66.05, 16.00 and 3.58%, respectively (**Table S1**). After alignment of the reads to the reference genome, variant calling and filtering, we identified a final set of 2,582,180 high-quality single-nucleotide polymorphisms (SNPs) and 412,999 indels (ranging from 1 to 244 bp in length) (**Table S1**). The distribution of variants across the genome was variable, depending on genome context and gene density (**Figure 1B**). A total of 69,992 SNPs (2.71%) and 3,652 indels (0.88%) were located in coding regions, among which 4,259 showed potentially large effects: 1,597 SNPs affected 1,152 genes by causing start codon changes, premature stop codons or elongated transcripts, and 2,662 indels led to frame shifts, gain of stop codons, or other disruptions of protein-coding capacity in 1,549 annotated genes (**Tables S2** and **S3**). The ratio of non-synonymous to synonymous SNPs (*N*/*S*) and transition to transversion SNPs (Ts/Tv) is 0.74 and 1.95, respectively (**Table S2**). We identified 27 genomic regions containing 673 genes with *N*/*S* ratio > 2.5 in all accessions (**Tables S4** and **S5**). Overall, we have generated a comprehensive mungbean genome variation dataset in which we identified numerous relevant SNPs and indels from diverse landraces.

### GWAS results

**Figure 2:**
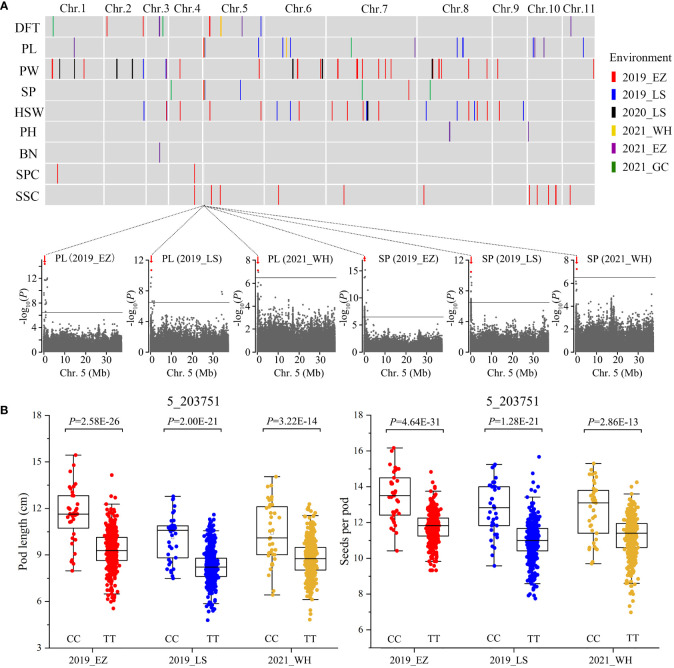
GWAS for nine agronomic traits and identification of the *SP5* locus on chromosome 5. **(A)** The distribution of the associated loci for nine agronomic traits in mungbean. Differently colored vertical lines indicate the associated loci for each trait: days to flowering time (DFT), pod length (PL), pod width (PW), seeds per pod (SP), 100-seed weight (HSW), plant height (PH), branch number (BN), seed protein content (SPC), and seed starch content (SSC). In the plots below, horizontal solid lines indicate the significance threshold (*P* < 3.87E-07, –log_10_ *P =* 6.41). Red arrows indicate strongly associated loci for both PL and SP at 0.16-0.28 Mb on chromosome 5, designated as *SEEDS PER POD ON CHROMOSOME 5* (*SP5*). The red dot indicates the peak SNP (5_203751, CC/TT). **(B)** Box plots illustrating PL and SP for the CC and TT alleles. Statistical significance for each environment was determined by a two-tailed *t*-test.

**Figure 3:**
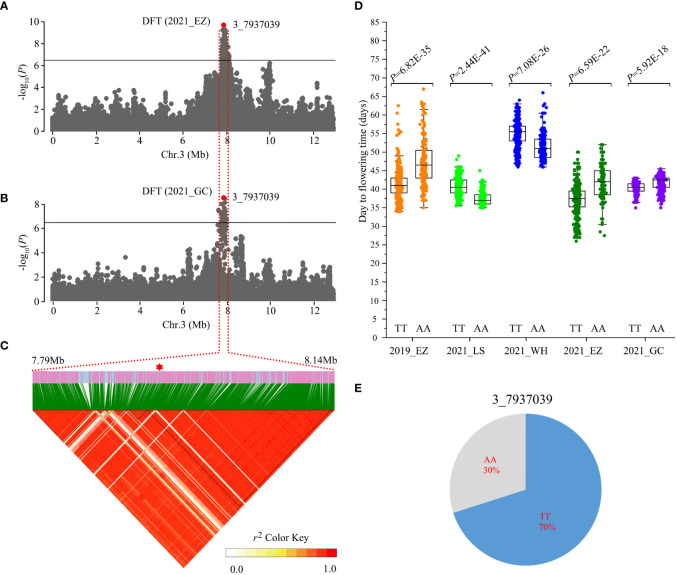
Identification of Chr3_7937039 flowering time loci. **(A, B)** Local Manhattan plots showing the number of days to flowering time (DFT) in the 2021_EZ **(A)** and 2021_GC environments **(B)**. Horizontal solid lines indicate the significance threshold (*P* < 3.87E-07, –log_10_ *P* = 6.41). The red dot indicates the peak SNP (3_7937039, T/A). **(C)** Linkage disequilibrium (LD) heatmap surrounding the peak SNP. **(D)** Box plots of DFT between the AA and TT alleles in different environments. Statistical significance for each environment was determined by a two-tailed *t*-test. **(E)** Genotype frequencies at the 3_7937039 SNP in the 558 accessions.

**Figure 4:**
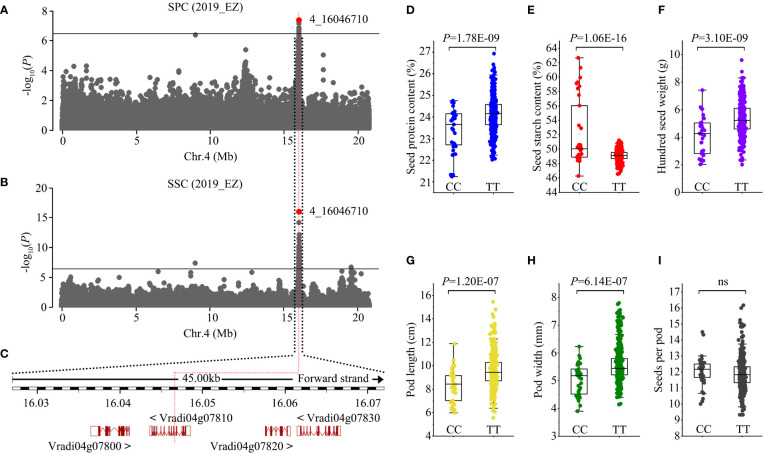
Identification of Chr4_16046710 seed protein content loci. **(A, B)** Manhattan plots of GWAS on seed protein content (SPC) and seed starch content (SSC) in the population. Horizontal solid lines indicate the significance threshold (*P* < 3.87E-07, –log_10_ *P* = 6.41). The red dot indicates the peak SNP (4_16046710, C/T). **(C)** Genes in the region associated with the peak SNP. The peak SNP is located at the ninth intron of *Vradi04g07810*. **(D-I)** Box plots of seed protein content **(D)**, seed starch content **(E)**, 100-seed weight **(F)**, pod length **(G)**, pod width **(H)**, and seeds per pod **(I)** for the two haplotypes. Statistical significance for each trait was determined by a two-tailed *t*-test. ns, no significant.

We measured nine traits in the 558 accessions from four agroecologically diverse locations, ranging from Mid-China to southern China, in 2019-2021 (**Table S6**), although because of resource constraints, we did not measure all nine traits in all six locations. These traits were days to flowering time (DFT), pod length (PL), pod width (PW), seeds per pod (SP), 100-seed weight (HSW), plant height (PH), branch number (BN), seed protein content (SPC), and seed starch content (SSC), all of which are crucial for the improvement of mungbean yield and end use. We observed diverse phenotypic variations for these traits (**Table S7**). Based on the 2,582,180 SNPs identified, we used a total of 37 sets of phenotypes assessed in six environments to perform GWAS using the genome-wide efficient mixed-model association (GEMMA; Zhou and Stephens, 2012) method. Manhattan plots and quantile-quantile plots of all nine traits from varied environments are shown in **Figures S1**-**S8**. In total, we identified 110 significant association signals (*P* < 3.87E-07, –log_10_ *P =* 6.41) for the nine traits in the mungbean genome (**Figure 2A** and **Table S8**). Among them, 12 association signals for the same traits were shared between at least two phenotyping environments (**Table S8**). Only a few candidate genes underlying agronomic traits have been identified in mungbean so far; thus, we integrated the GWAS approach with functional annotation of the orthologs in model plants to rapidly identify candidate genes associated with seeds per pod, pod length (**Figure 2**), days to flowering time (**Figure 3**), and seed protein content (**Figure 4**).

Seeds per pod is one of the main determinants of seed yield in mungbean and is positively correlated with pod length. Out of 110 GWAS signals for all nine traits, 10 and 18 signals were associated loci for seeds per pod and pod length, respectively. We identified a major locus responsible for both seeds per pod and pod length at Mb 0.16-0.28 on chromosome 5 in three environments (**Figure 2A**), which we designated *SEEDS PER POD ON CHROMOSOME 5* (*SP5*). Based on the peak SNP (5_203751, C/T) of the association signal, two haplotypes were identified in all 558 accessions. Accessions carrying the CC allele exhibited increased seeds per pod and pod length compared to accessions carrying the TT allele (~14.38% and ~21.64% greater SP and PL, respectively) (**Figure 2B**). However, pod width and 100-seed weight did not significantly differ between accessions with CC vs. TT alleles. After carefully analyzing the 13 genes in this region of chromosome 5 (**Table S9**), we identified a candidate gene encoding a leucine-rich repeat serine-threonine/tyrosine-protein kinase (*Vradi05g00200*). Leucine-rich-repeat receptor-like kinases are involved in polar auxin transport in plants (Afzal et al., 2008; Zou et al., 2014), and auxin regulates the silique length of rapeseed and the kernel number per row of maize (Liu et al., 2015; Jia et al., 2020; Li et al., 2021). We therefore propose *Vradi05g00200* as the key candidate gene for *SP5*.

The number of days to flowering time is critical for modern crop production and a major trait associated with crop adaptation. This trait has been reported to be highly sensitive to environmental temperature and photoperiod in crops (Hung et al., 2012; Lu et al., 2020; Wei et al., 2020). In the present study, we identified a strong GWAS signal for days to flowering time at Mb 7.79-8.14 on chromosome 3 using phenotype data from 2021_EZ and 2021_GC (**Figures 3A, B**). This GWAS signal showed weak associations in the other environments (**Figure S1**). The ~200 kb (Mb 7.93-8.13) LD block surrounding the peak SNP (3_7937039, T/A) contains 22 gene models (**Figure 3C**, **Table S10**). The 3_7937039 SNP generated two haplotypes, TT and AA, and resides 5 kb downstream of *Vradi03g06500*, a gene encoding a Calvin cycle protein, CP12-2, whose orthologue in Arabidopsis regulates flowering time (Singh et al., 2008; Elena Lóez-Calcagno et al., 2017). The peak SNP 3_7937039 exhibited an opposite direction of effect between accessions carrying the TT allele and AA allele in different environments (**Figure 3D**). Landraces carrying the TT allele showed significantly earlier flowering time than those carrying the AA allele under long day conditions (2019_EZ, 2021_EZ and 2021_GC), but significantly later flowering in environments with short days (2020_LS and 2021_WH). Through further analyses assessing the frequency of different haplotypes in the 558 accessions, we found that 70% of landraces carried the TT genotype, while 30 of landraces carried the AA genotype (**Figure 3E**).These results suggest that *Vradi03g06500* may be a strong candidate for the flowering time locus.

Protein and starch are the two most abundant components of mungbean seeds. GWAS results showed that both SPC and SSC were associated with SNPs in one genomic region ranging from Mb 16.027 to 16.073 on chromosome 4 (**Figure 4A, B**). Four candidate genes, *Vradi04g07800*, *Vradi04g07810*, *Vradi04g07820*, and *Vradi04g07830*, were found in this association region, and the peak SNP (4_16046710, C/T) was located within the ninth intron of *Vradi04g07810* (**Figure 4C**). *Vradi04g07810* encodes a serine carboxypeptidase whose orthologues in Arabidopsis and tobacco mediate brassinosteroid signaling and has an impact on cell elongation (Li et al., 2001; Bienert et al., 2012). The peak SNP generated two haplotypes: CC and TT. Accessions carrying the TT allele had significantly higher SPC but lower SSC than those with the CC allele (**Figure 4D, E**). Additionally, we found that the TT allele correlated with larger pod size (PL × PW) and higher HSW, and that the CC allele correlated with smaller pod size and lower HSW (**Figure 4F-I**). Given that protein and starch account for ~80% of mungbean seed content, it is reasonable that genes regulating both SPC and SSC will also influence seed weight. These results indicate that *Vradi04g07810* could be a candidate gene for this pleiotropic locus.

## Discussion

Landraces in national and international germplasm banks provide a rich source of genetic diversity that may be vital for future crop improvement. However, less than 2% of these germplasm resources have been utilized in modern crop breeding programs (Varshney et al., 2020). One of the reasons for the limited use of such germplasm collections is that for the vast majority of these accessions, no phenotypic or genotypic information is available. With recent advances in next-generation sequencing (NGS) technology and significant reductions in the cost of genome sequencing, it is now possible to sequence large-scale collections of crop accessions. But field-based phenotyping is a bottleneck in the characterization of large-scale crop accessions because it is time and resource intensive. A total of 1,038 mungbean accessions were investigated by GBS in three recent studies (Noble et al., 2017; Breria et al., 2020; Ha et al., 2021). These studies provide genome-wide variant information and insights on the population structure of mungbean accessions. However, to more fully mine valuable genetic information requires performing phenotypic analyses of the large-scale collections. In this study, our resequencing of 558 representative landraces, which were selected to represent much of the phenotypic and geographic diversity of the Chinese mungbean collection, yielded 2.58 million high-quality SNPs and a comprehensive genome variation map of mungbean. Moreover, we planted and phenotyped all 558 accessions in four field locations ranging from mid to southern China in 2019-2021. From these analyses, 37 sets of phenotypes were obtained. By combining genotypic and phenotypic data from the 558 accessions, we performed the first GWAS analysis for agronomic traits in mungbean. Overall, this study lays the foundation for a long-term collective effort to develop improved mungbean strains by discovering valuable genes and alleles from worldwide germplasm collections.

Mungbean landraces have evolved from their wild progenitor under natural and human selection, leading to the maintenance of high genetic diversity. Identifying the genetic basis of these landraces will provide important insights necessary to breed elite mungbean varieties for modern agriculture. GWAS has become a routine approach to decode genotype-phenotype associations in many crop species thanks to advances in NGS technologies (Liu and Yan, 2019). A comprehensive map of genomic variations is essential to identify additional QTLs/genes associated with traits from the GWAS analysis in crops. Two previous studies performed association analyses of seed coat color and luster using a limited number of SNPs from GBS in mungbean; however, only a few markers were identified to be associated with these two traits at a low statistical level (Noble et al., 2017; Breria et al., 2020). Adding to these data, our study provides 2.58 million high-quality SNPs and a comprehensive genome variation map of mungbean. Identification of these variations facilitates comprehensive association analyses of quantitative traits in mungbean. We discovered 110 SNPs associated with nine agronomic traits and identified gene candidates for several of these traits. Although genomics and genetics have greatly accelerated the dissection of potential genes or networks related to crop traits in the past two decades, there remain many limitations that hinder further causative gene identification and gene function verification. For instance, an incomplete reference genome (Ha et al., 2021) and immature transgenic technology are two major obstacles to assigning gene function in mungbean. Therefore, further work is necessary to identify specific genes underlying agronomic traits. Collectively, the results of this study provide insights into the genetic architecture of mungbean agronomic traits, and the genome-wide variations identified are valuable for future breeding studies on this food legume.

## Data availability statement

The data presented in the study are deposited in the NCBI repository, accession number PRJNA885164 (SRR21783999 - SRR21784556).

## Author contributions

ZW, CL and XH conceived and designed the experiments. XH, XW, YX, LiL, LiaL, HC and LS performed the experiments. XH analysed data and wrote the manuscript. All authors contributed to the article and approved the submitted version.

## Funding

This work was supported in part by the National Key Research and Development Program of China (2019YFD1001303 and 2019YFD1001300), China Agriculture Research System (CARS-08), and the National Natural Science Foundation of China (32101808).

## Acknowledgments

We thank Professor Xin Chen (Institute of Industrial Crops, Jiangsu Academy of Agricultural Sciences, Nanjing, China), Professor Jing Tian (Institute of Cereal and Oil Crops, Hebei Academy of Agricultural and Forestry Sciences, Shijiazhuang, China.), Professor Bin Zhou (Crop Institute of Anhui Academy of Agricultural Sciences, Hefei, China), Professor Xu Zhu (Nanyang Academy of Agricultural Sciences, Nanyang, China), Professor Yanlan Wang (Hunan Crop Research Institute, Changsha, China), and Professor Huijun Zhu (College of Agronomy, Shanxi Agricultural University, Taigu, China) for providing part of mungbean accessions used in the study.

## Conflict of interest

The authors declare that the research was conducted in the absence of any commercial or financial relationships that could be construed as a potential conflict of interest.

## Publisher’s note

All claims expressed in this article are solely those of the authors and do not necessarily represent those of their affiliated organizations, or those of the publisher, the editors and the reviewers. Any product that may be evaluated in this article, or claim that may be made by its manufacturer, is not guaranteed or endorsed by the publisher.

## References

[B1] Afzal A. J. Wood A. J. Lightfoot D. A. (2008). Plant receptor-like serine threonine kinases: Roles in signaling and plant defense. Mol. Plant Microbe Interact. 21, 507–517. doi: 10.1094/MPMI-21-5-0507 18393610

[B2] Bienert M. D. Delannoy M. Navarre C. Boutry M. (2012). NtSCP1 from tobacco is an extracellular serine carboxypeptidase III that has an impact on cell elongation. Plant Physiol. 158, 1220–1229. doi: 10.1104/pp.111.192088 22214816 PMC3291266

[B3] Breria C. M. Hsieh C. H. Yen J. Y. Nair R. Lin C. Y. Huang S. M.. (2020). Population structure of the world vegetable center mungbean mini core collection and genome-wide association mapping of loci associated with variation of seed coat luster. Trop. Plant Biol. 13, 1–12. doi: 10.1007/s12042-019-09236-0

[B4] Browning B. L. Zhou Y. Browning S. R. (2018). A one-penny imputed genome from next-generation reference panels. Am. J. Hum. Genet. 103, 338–348. doi: 10.1016/j.ajhg.2018.07.015 30100085 PMC6128308

[B5] Chang C. C. Chow C. C. Tellier L. C. Vattikuti S. Purcell S. M. Lee J. J. (2015). Second-generation PLINK: rising to the challenge of larger and richer datasets. Gigascience 4, 1–16. doi: 10.1186/s13742-015-0047-8 25722852 PMC4342193

[B6] Chen Y. Chen Y. Shi C. Huang Z. Zhang Y. Li S.. (2018). SOAPnuke: A MapReduce acceleration-supported software for integrated quality control and preprocessing of high-throughput sequencing data. Gigascience 7, 1–6. doi: 10.1093/gigascience/gix120 PMC5788068 29220494

[B7] Cheng X. Z. (2006). Descriptors and data standard for mungBean (Vicia radiatus l.) (Beijing: China Agricultural Press).

[B8] Chen D. H. Ronald P. C. (1999). A rapid DNA minipreparation method suitable for AFLP and other PCR applications. Plant Mol. Biol. Rep. 17, 53–57. doi: 10.1023/A:1007585532036

[B9] Elena López-Calcagno P. Omar Abuzaid A. Lawson T. Anne Raines C. (2017). Arabidopsis CP12 mutants have reduced levels of phosphoribulokinase and impaired function of the Calvin-Benson cycle. J. Exp. Bot. 68, 2285–2298. doi: 10.1093/jxb/erx084 28430985 PMC5447874

[B10] Fang C. Ma Y. Wu S. Liu Z. Wang Z. Yang R. (2017). Genome-wide association studies dissect the genetic networks underlying agronomical traits in soybean. Genome Biol. 18, 161. doi: 10.1186/s13059-017-1289-9 28838319 PMC5571659

[B11] Graham P. H. Vance C. P. (2003). Legumes: importance and constraints to greater use. Plant Physiol. 131, 872–877. doi: 10.1104/pp.017004 12644639 PMC1540286

[B12] Ha J. Satyawan D. Jeong H. Lee E. Cho K. H. Kim M. Y.. (2021). A near-complete genome sequence of mungbean (*Vigna radiata* l.) provides key insights into the modern breeding program. Plant Genome 14, e20121. doi: 10.1002/tpg2.20121 34275211 PMC12807378

[B13] Huang X. Wei X. Sang T. Zhao Q. Feng Q. Zhao Y.. (2010). Genome-wide association studies of 14 agronomic traits in rice landraces. Nat. Genet. 42, 961–967. doi: 10.1038/ng.695 20972439

[B14] Hung H. Y. Shannon L. M. Tian F. Bradbury P. J. Chen C. Flint-Garcia S. A.. (2012). ZmCCT and the genetic basis of day-length adaptation underlying the postdomestication spread of maize. Proc. Natl. Acad. Sci. U.S.A. 109, 1913–1921. doi: 10.1073/pnas.1203189109 22711828 PMC3396540

[B15] Isemura T. Kaga A. Tabata S. Somta P. Srinives P. Shimizu T.. (2012). Construction of a genetic linkage map and genetic analysis of domestication related traits in mungbean (*Vigna radiata*). PloS One 7, e41304. doi: 10.1371/journal.pone.0041304 22876284 PMC3410902

[B16] Jia H. Li M. Li W. Liu L. Jian Y. Yang Z.. (2020). A serine/threonine protein kinase encoding gene *KERNEL NUMBER PER ROW6* regulates maize grain yield. Nat. Commun. 11, 988. doi: 10.1038/s41467-020-14746-7 32080171 PMC7033126

[B17] Kang Y. J. Kim S. K. Kim M. Y. Lestari P. Kim K. H. Ha B. K.. (2014). Genome sequence of mungbean and insights into evolution within *Vigna* species. Nat. Commun. 5, 5443. doi: 10.1038/ncomms6443 25384727 PMC4241982

[B18] Kim S. K. Nair R. M. Lee J. Lee S. H. (2015). Genomic resources in mungbean for future breeding programs. Front. Plant Sci. 6, 626. doi: 10.3389/fpls.2015.00626 26322067 PMC4530597

[B19] Li X. Chen Z. Zhang G. Lu H. Qin P. Qi M.. (2020). Analysis of genetic architecture and favorable allele usage of agronomic traits in a large collection of Chinese rice accessions. Sci. China Life Sci. 63, 1688–1702. doi: 10.1007/s11427-019-1682-6 32303966

[B20] Li H. Durbin R. (2009). Fast and accurate short read alignment with burrows-wheeler transform. Bioinformatics 25, 1754–1760. doi: 10.1093/bioinformatics/btp324 19451168 PMC2705234

[B21] Li H. Handsaker B. Wysoker A. Fennell T. Ruan J. Homer N.. (2009). The sequence alignment/map format and SAMtools. Bioinformatics 25, 2078–2079. doi: 10.1093/bioinformatics/btp352 19505943 PMC2723002

[B22] Li J. Lease K. A. Tax F. E. Walker J. C. (2001). BRS1, a serine carboxypeptidase, regulates BRI1 signaling in *Arabidopsis thaliana* . Proc. Natl. Acad. Sci. U.S.A. 98, 5916–5921. doi: 10.1073/pnas.091065998 11320207 PMC33313

[B23] Liu J. Hua W. Hu Z. Yang H. Zhang L. Li R.. (2015). Natural variation in ARF18 gene simultaneously affects seed weight and silique length in polyploid rapeseed. Proc. Natl. Acad. Sci. U.S.A. 112, 5123–5132. doi: 10.1073/pnas.1502160112 26324896 PMC4577148

[B24] Liu C. Wang Y. Peng J. Fan B. Xu D. Wu J.. (2022). High-quality genome assembly and pan-genome studies facilitate genetic discovery in mung bean and its improvement. Plant Commun. 100352. doi: 10.1016/j.xplc.2022.100352 35752938 PMC9700124

[B25] Liu H. J. Yan J. (2019). Crop genome-wide association study: A harvest of biological relevance. Plant J. 97, 8–18. doi: 10.1111/tpj.14139 30368955

[B26] Li M. Zhao R. Du Y. Shen X. Ning Q. Li Y.. (2021). The coordinated KNR6-AGAP-ARF1 complex modulates vegetative and reproductive traits by participating in vesicle trafficking in maize. Cells 10, 2601. doi: 10.3390/cells10102601 34685581 PMC8533723

[B27] Lu S. Dong L. Fang C. Liu S. Kong L. Cheng Q.. (2020). Stepwise selection on homeologous PRR genes controlling flowering and maturity during soybean domestication. Nat. Genet. 52, 428–436. doi: 10.1038/s41588-020-0604-7 32231277

[B28] Ma Z. He S. Wang X. Sun J. Zhang Y. Zhang G.. (2018). Resequencing a core collection of upland cotton identifies genomic variation and loci influencing fiber quality and yield. Nat. Genet. 50, 803–813. doi: 10.1038/s41588-018-0119-7 29736016

[B29] McKenna A. Hanna M. Banks E. Sivachenko A. Cibulskis K. Kernytsky A.. (2010). The genome analysis toolkit: a MapReduce framework for analyzing next-generation DNA sequencing data. Genome Res. 20, 1297–1303. doi: 10.1101/gr.107524.110 20644199 PMC2928508

[B30] Nair R. M. Yang R. Y. Easdown W. J. Thavarajah D. Thavarajah P. Hughes J.. (2013). Biofortification of mungbean (*Vigna radiata*) as a whole food to enhance human health. J. Sci. Food Agric. 93, 1805–1813. doi: 10.1002/jsfa.6110 23426879

[B31] Noble T. J. Tao Y. Mace E. S. Williams B. Jordan D. R. Douglas C. A.. (2017). Characterization of linkage disequilibrium and population structure in a mungbean diversity panel. Front. Plant Sci. 8, 2102. doi: 10.3389/fpls.2017.02102 29375590 PMC5770403

[B32] Schafleitner R. Nair R. M. Rathore A. Wang Y. W. Lin C. Y. Chu S. H.. (2015). The AVRDC - the world vegetable center mungbean (*Vigna radiata*) core and mini core collections. BMC Genomics 16, 344. doi: 10.1186/s12864-015-1556-7 25925106 PMC4422537

[B33] Singh P. Kaloudas D. Raines C. A. (2008). Expression analysis of the arabidopsis CP12 gene family suggests novel roles for these proteins in roots and floral tissues. J. Exp. Bot. 59, 3975–3985. doi: 10.1093/jxb/ern236 18974062 PMC2576635

[B34] Smýkal P. Nelson M. N. Berger J. D. von Wettberg E. J. B. (2018). The impact of genetic changes during crop domestication. Agronomy 8, 119.

[B35] Turner S. D. (2014). Qqman: an r package for visualizing GWAS results using q-qand manhattan plots. BioRxiv. doi: 10.1101/005165

[B36] Varshney R. K. Saxena R. K. Upadhyaya H. D. Khan A. W. Yu Y. Kim C.. (2017). Whole-genome resequencing of 292 pigeonpea accessions identifies genomic regions associated with domestication and agronomic traits. Nat. Genet. 49, 1082–1088. doi: 10.1038/ng.3872 28530677

[B37] Varshney R. K. Sinha P. Singh V. K. Kumar A. Zhang Q. Bennetzen J. L. (2020). 5Gs for crop genetic improvement. Curr. Opin. Plant Biol. 56, 190–196. doi: 10.1016/j.pbi.2019.12.004 32005553 PMC7450269

[B38] Varshney R. K. Thudi M. Roorkiwal M. He W. Upadhyaya H. D. Yang W.. (2019). Resequencing of 429 chickpea accessions from 45 countries provides insights into genome diversity, domestication and agronomic traits. Nat. Genet. 51, 857–864. doi: 10.1038/s41588-019-0401-3 31036963

[B39] Wang K. Li M. Hakonarson H. (2010). ANNOVAR: functional annotation of genetic variants from high-throughput sequencing data. Nucleic Acids Res. 38, e164. doi: 10.1093/nar/gkq603 20601685 PMC2938201

[B40] Wang B. Lin Z. Li X. Zhao Y. Zhao B. Wu G.. (2020). Genome-wide selection and genetic improvement during modern maize breeding. Nat. Genet. 52, 565–571. doi: 10.1038/s41588-020-0616-3 32341525

[B41] Wei H. Wang X. Xu H. Wang L. (2020). Molecular basis of heading date control in rice. aBIOTECH 1, 219–232. doi: 10.1007/s42994-020-00019-w PMC9590479 36304129

[B42] Wu J. Wang L. Fu J. Chen J. Wei S. Zhang S.. (2020). Resequencing of 683 common bean genotypes identifies yield component trait associations across a north-south cline. Nat. Genet. 52, 118–125. doi: 10.1038/s41588-019-0546-0 31873299

[B43] Xiao Y. Liu H. Wu L. Warburton M. Yan J. (2017). Genome-wide association studies in maize: praise and stargaze. Mol. Plant 10, 359–374. doi: 10.1016/j.molp.2016.12.008 28039028

[B44] Zhou X. Stephens M. (2012). Genome-wide efficient mixed-model analysis for association studies. Nat. Genet. 44, 821–824. doi: 10.1038/ng.2310 22706312 PMC3386377

[B45] Zou Y. Liu X. Wang Q. Chen Y. Liu C. Qiu Y.. (2014). OsRPK1, a novel leucine-rich repeat receptor-like kinase, negatively regulates polar auxin transport and root development in rice. Biochim. Biophys. Acta 1840, 1676–1685. doi: 10.1016/j.bbagen.2014.01.003 24412327

