# Supplementary material for: Resequencing of 558 Chinese mungbean landraces identifies genetic loci associated with key agronomic traits
Source: Front Plant Sci. 2022 Oct 12;13:1043784. doi: 10.3389/fpls.2022.1043784 (PMC9597495; doi:10.3389/fpls.2022.1043784)
Supplement: Supplementary file 1 [file DataSheet_1.pdf]

Supplementary Figures

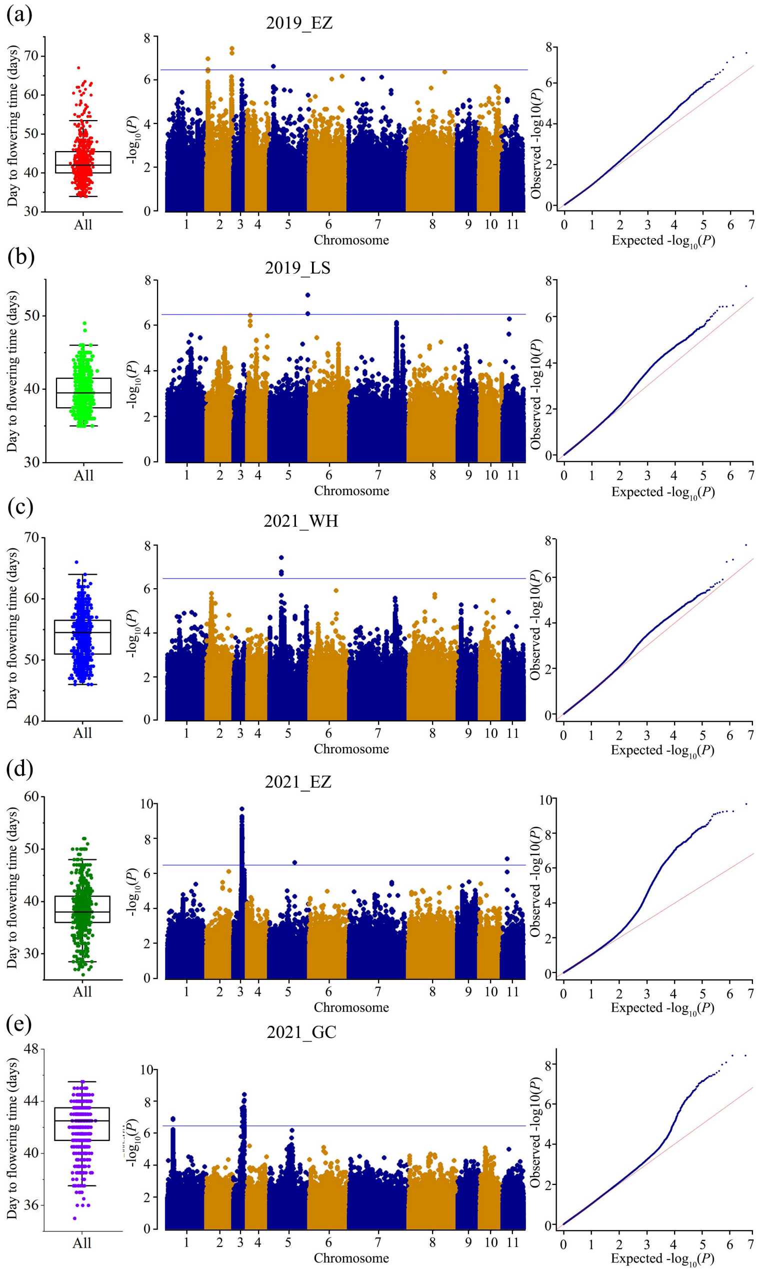

Figure S1 GWAS analysis of days to flowering time in five environments. (a) Plants grown in Ezhou in 2019 (2019_EZ). (b) Plants grown in Lingshui in 2019 (2019_LS). (c) Plants grown in Wuhan in 2021 (2021_WH). (d) Plants grown in Ezhou in 2021 (2021_EZ). (e) Plants grown in Gucheng in 2021 (2021_GC). Left, box plots illustrating the number of days to flowering time for all landraces. Middle, Manhattan plots of GWAS data. The significance threshold of $-\log_{10} P$ was set at 6.41. Right, quantile-quantile plots of days to flowering time.

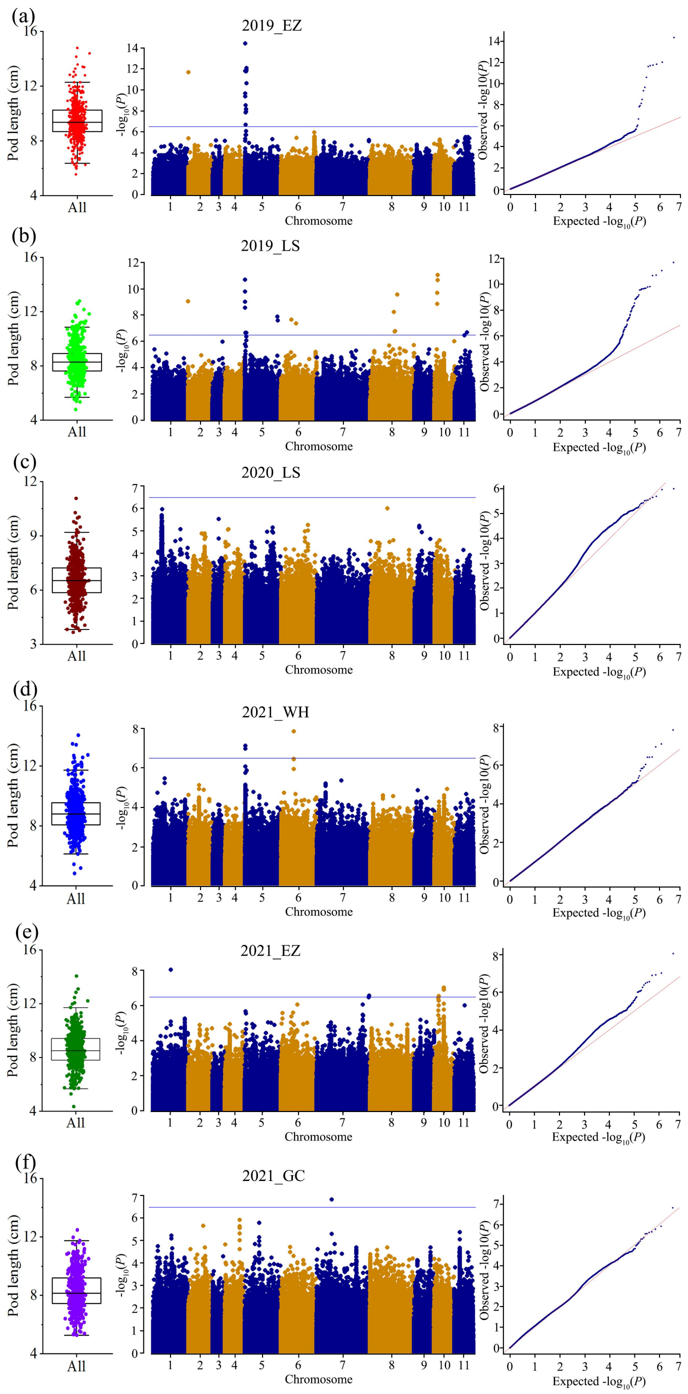

Figure S2 GWAS analysis of pod length in six environments. (a) Plants grown in Ezhou in 2019 (2019_EZ). (b) Plants grown in Lingshui in 2019 (2019_LS). (c) Plants grown in Lingshui in 2020 (2020_LS). (d) Plants grown in Wuhan in 2021 (2021_WH). (e) Plants grown in Ezhou in 2021 (2021_EZ). (f) Plants grown in Gucheng in 2021 (2021_GC). Left, box plots showing pod length for all landraces. Middle, Manhattan plots of GWAS data. The significance threshold of $-\log_{10} P$ was set at 6.41. Right, quantile-quantile plots of pod length.

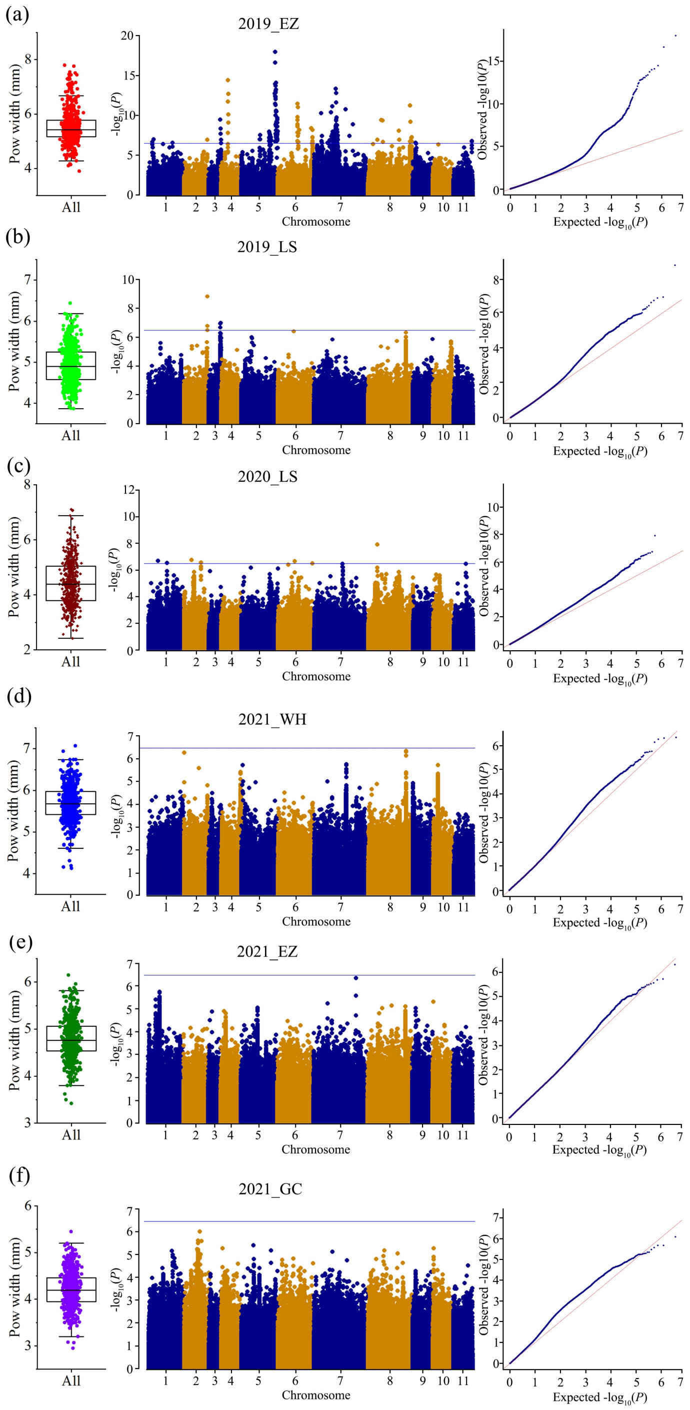

Figure S3 GWAS analysis of pod width in six environments. (a) Plants grown in Ezhou in 2019 (2019_EZ). (b) Plants grown in Lingshui in 2019 (2019_LS). (c) Plants grown in Lingshui in 2020 (2020_LS). (d) Plants grown in Wuhan in 2021 (2021_WH). (e) Plants grown in Ezhou in 2021 (2021_EZ). (f) Plants grown in Gucheng in 2021 (2021_GC). Left, box plots demonstrating pod width of all landraces. Middle, Manhattan plots of GWAS data. The significance threshold of $-\log_{10} P$ was set at 6.41. Right, quantile-quantile plots of pod width.

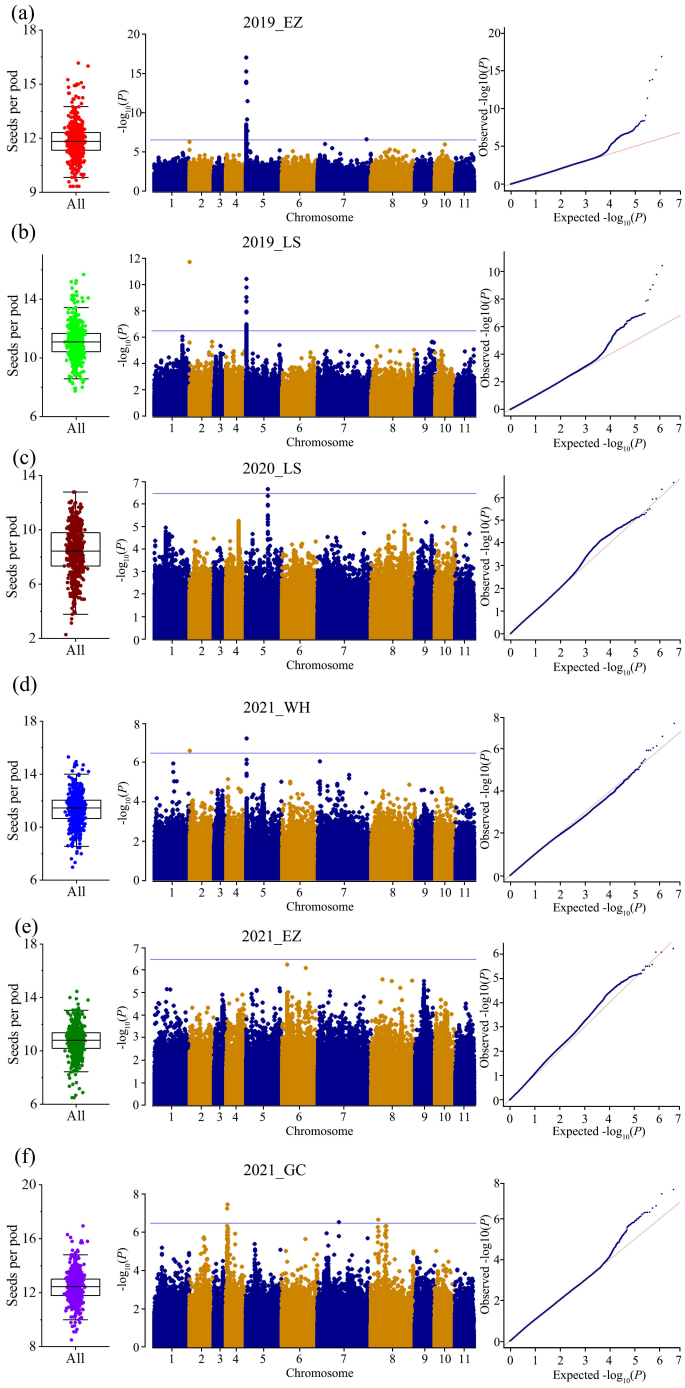

Figure S4 GWAS analysis of seeds per pod in six environments. (a) Plants grown in Ezhou in 2019 (2019_EZ). (b) Plants grown in Lingshui in 2019 (2019_LS). (c) Plants grown in Lingshui in 2020 (2020_LS). (d) Plants grown in Wuhan in 2021 (2021_WH). (e) Plants grown in Ezhou in 2021 (2021_EZ). (f) Plants grown in Gucheng in 2021 (2021_GC). Left, box plots showing the number of seeds per pod for all landraces. Middle, Manhattan plots of GWAS data. The significance threshold of $-\log_{10} P$ was set at 6.41. Right, quantile-quantile plots of seeds per pod.

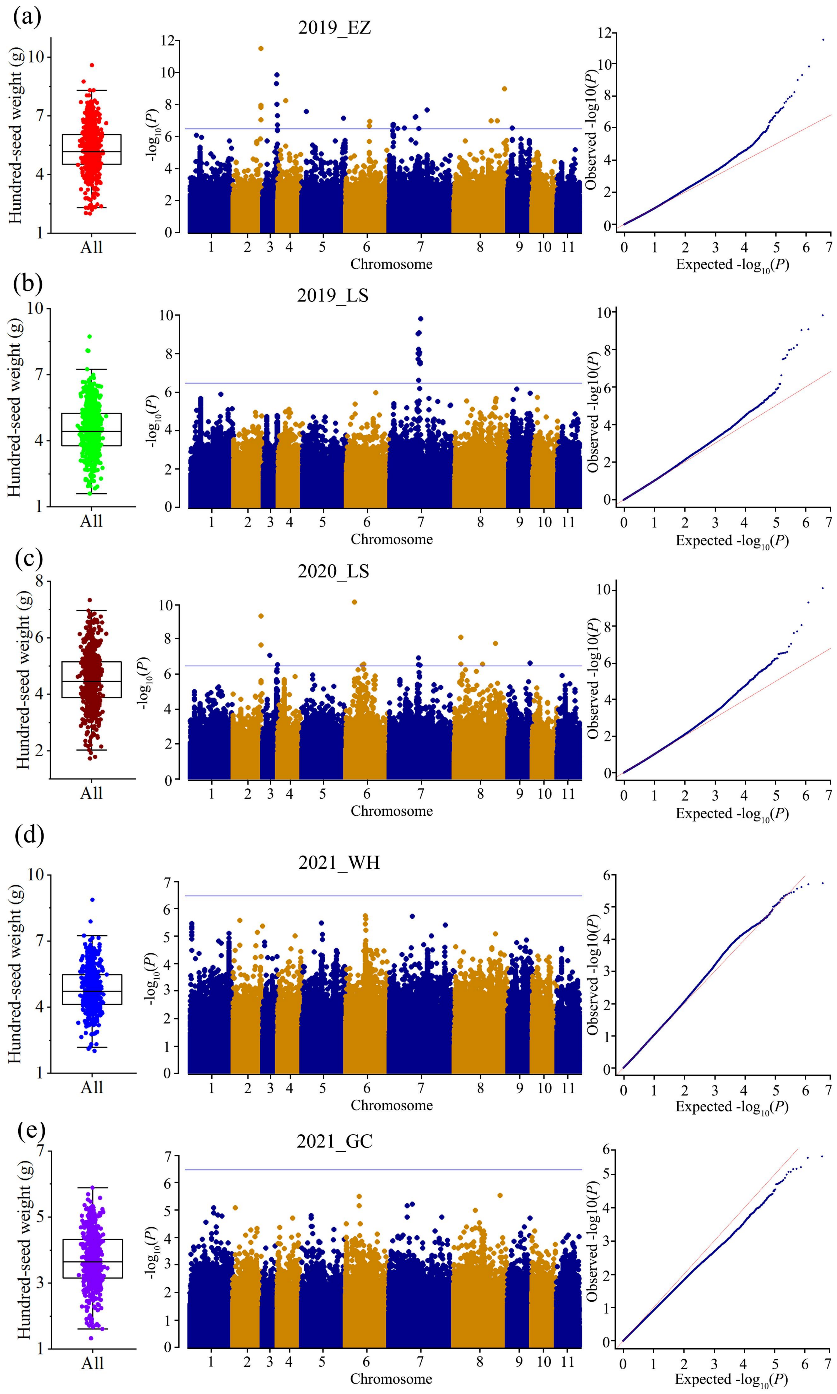

Figure S5 GWAS analysis of hundred-seed weight in five environments. (a) Plants grown in Ezhou in 2019 (2019_EZ). (b) Plants grown in Lingshui in 2019 (2019_LS). (c) Plants grown in Lingshui in 2020 (2020_LS). (d) Plants grown in Wuhan in 2021 (2021_WH). (e) Plants grown in Gucheng in 2021 (2021_GC). Left, box plots illustrating the hundred-seed weight for all landraces. Middle, Manhattan plots of GWAS data. The significance threshold of $-\log_{10} P$ was set at 6.41. Right, quantile-quantile plots of hundred-seed weight.

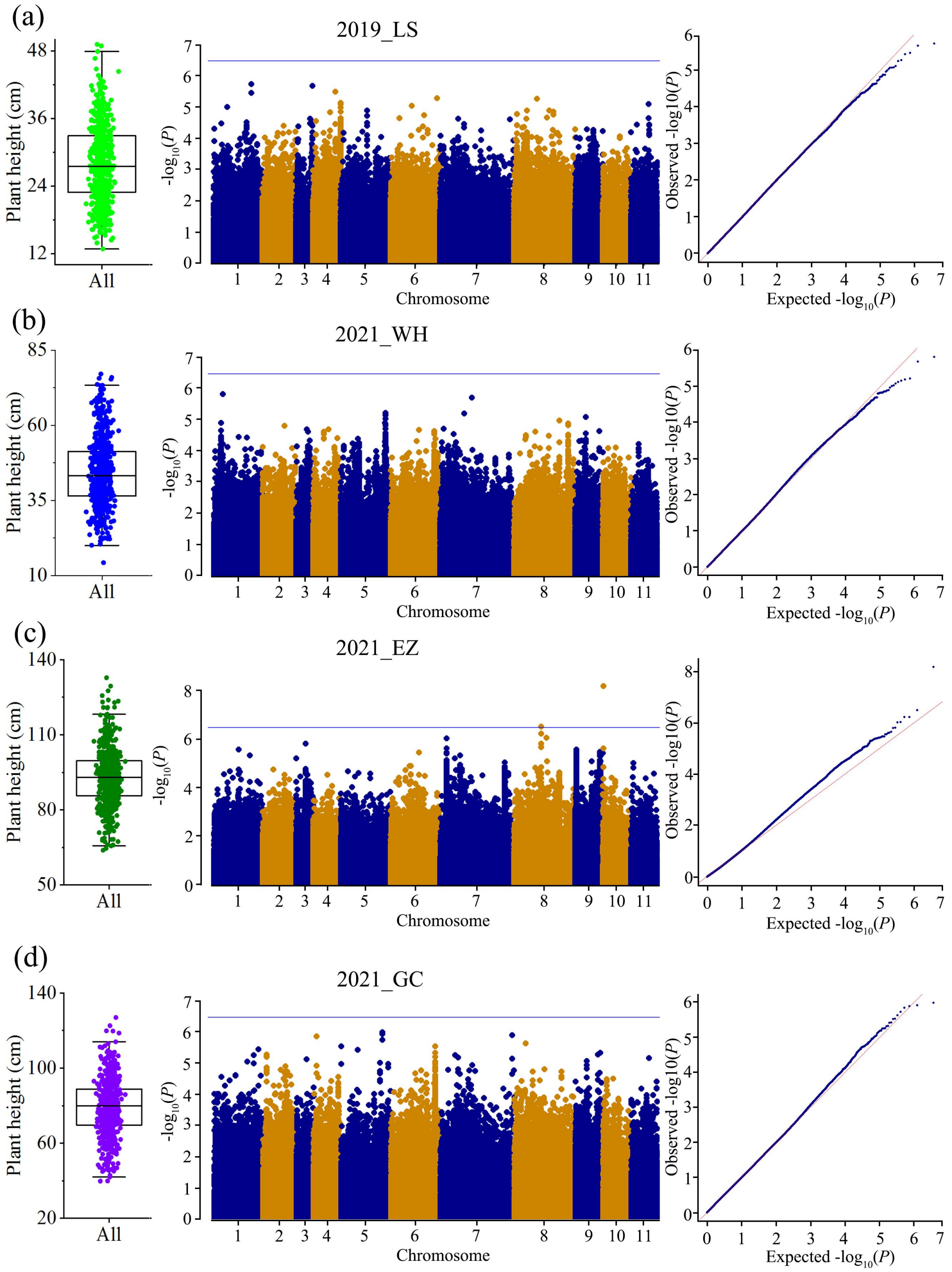

Figure S6 GWAS analysis of plant height in four environments. (a) Plants grown in Ezhou in 2019 (2019_EZ). (b) Plants grown in Wuhan in 2021 (2021_WH). (c) Plants grown in Ezhou in 2021 (2021_EZ). (d) Plants grown in Gucheng in 2021 (2021_GC). Left, box plots demonstrating plant height for all landraces. Middle, Manhattan plots of GWAS data. The significance threshold of $-\log_{10} P$ was set at 6.41. Right, quantile-quantile plots of plant height.

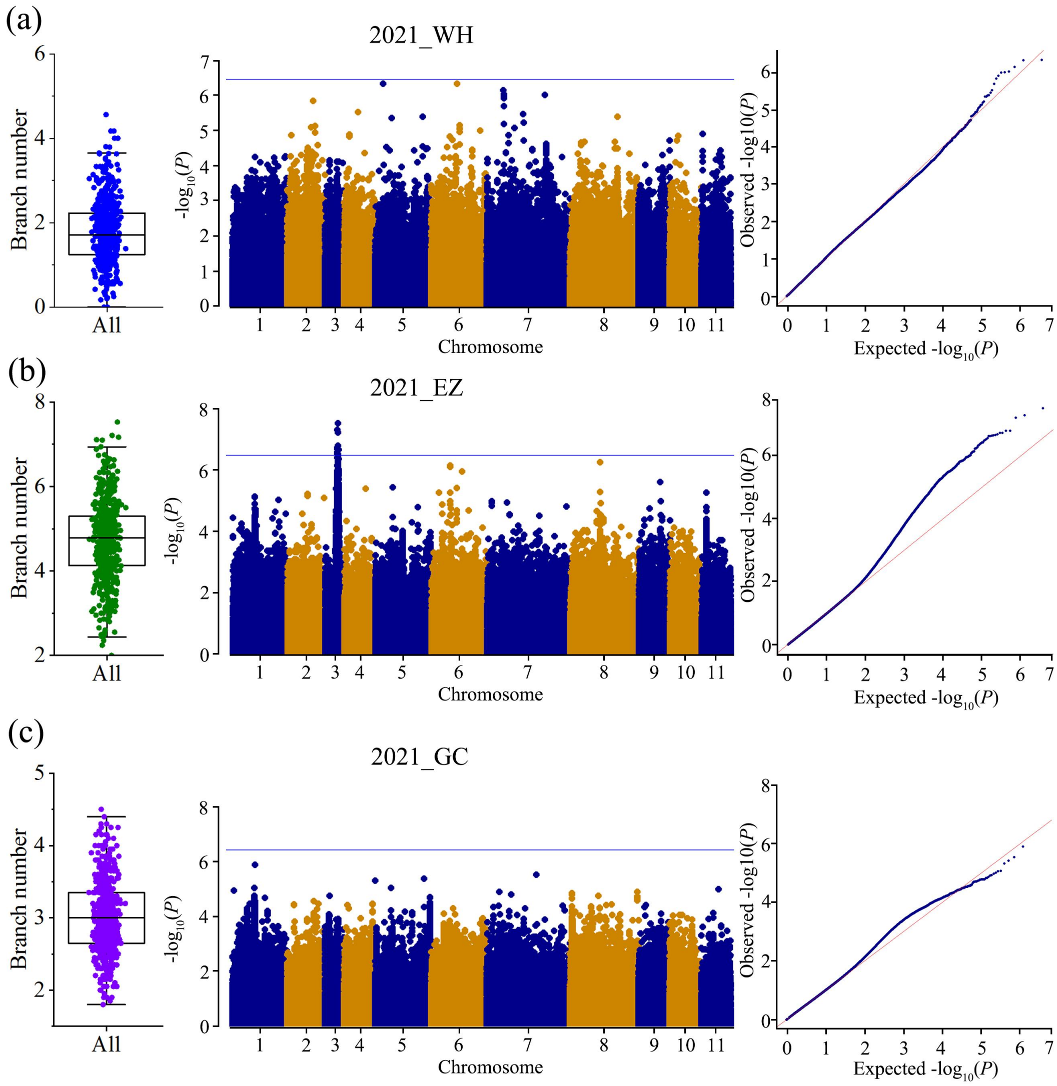

Figure S7 GWAS analysis of branch number in three environments. (a) Plants grown in Wuhan in 2021 (2021_WH). (b) Plants grown in Ezhou in 2021 (2021_EZ). (c) Plants grown in Gucheng in 2021 (2021_GC). Left, box plots illustrating branch number for all landraces. Middle, Manhattan plots of GWAS data. The significance threshold of $-\log_{10} P$ was set at 6.41. Right, quantile-quantile plots of branch number.

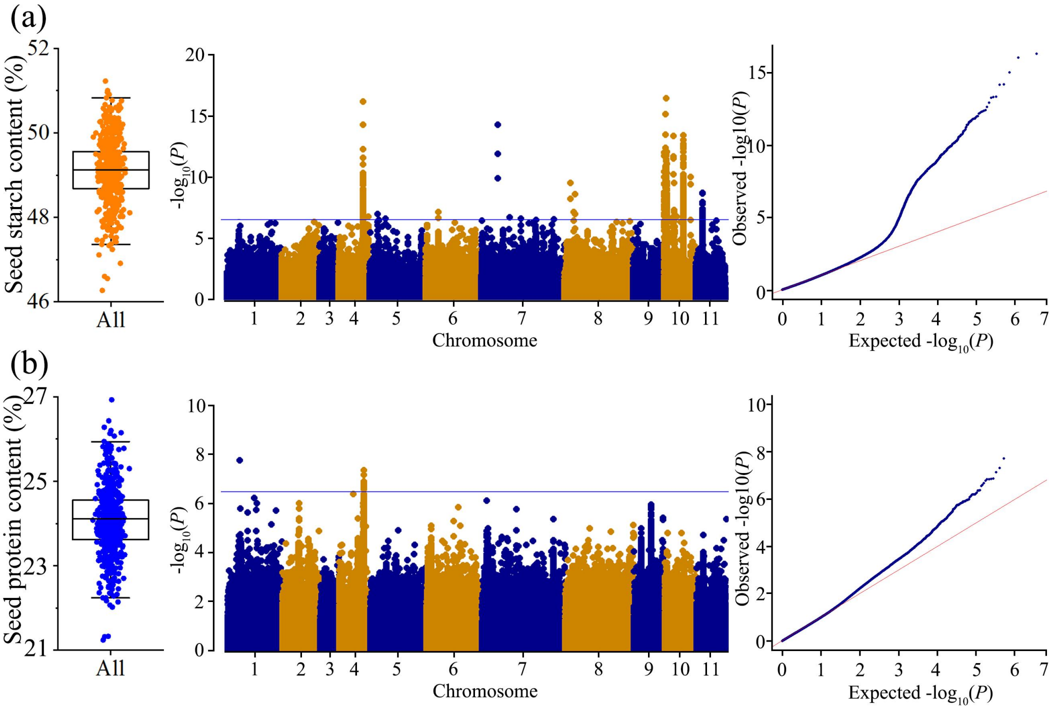

Figure S8 GWAS analysis of seed starch content and seed protein content. (a) GWAS analysis of seed starch content. Left, box plots showing seed starch content for all landraces. Middle, Manhattan plots of GWAS data. The significance threshold of $-\log_{10} P$ was set at 6.41. Right, quantile-quantile plots of seed starch content. (b) GWAS analysis of seed protein content. Left, box plots showing seed protein content for all landraces. Middle, Manhattan plots of GWAS data. The significance threshold of $-\log_{10} P$ was set at 6.41. Right, quantile-quantile plots of seed protein content.
